# Does the Nutritional Composition of Dairy Milk Based Recovery Beverages Influence Post-exercise Gastrointestinal and Immune Status, and Subsequent Markers of Recovery Optimisation in Response to High Intensity Interval Exercise?

**DOI:** 10.3389/fnut.2020.622270

**Published:** 2021-01-14

**Authors:** Isabella Russo, Paul A. Della Gatta, Andrew Garnham, Judi Porter, Louise M. Burke, Ricardo J. S. Costa

**Affiliations:** ^1^Department of Nutrition Dietetics & Food, Monash University, Notting Hill, VIC, Australia; ^2^School of Exercise and Nutrition Sciences, Institute for Physical Activity and Nutrition, Deakin University, Geelong, VIC, Australia; ^3^Mary MacKillop Institute for Health Research, Australian Catholic University, Melbourne, VIC, Australia

**Keywords:** molecular nutrition, intestinal epithelium, neutrophil, inflammation, glycogen, protein synthesis, hydration

## Abstract

This study aimed to determine the effects of flavored dairy milk based recovery beverages of different nutrition compositions on markers of gastrointestinal and immune status, and subsequent recovery optimisation markers. After completing 2 h high intensity interval running, participants (*n* = 9) consumed a whole food dairy milk recovery beverage (CM, 1.2 g/kg body mass (BM) carbohydrate and 0.4 g/kg BM protein) or a dairy milk based supplement beverage (MBSB, 2.2 g/kg BM carbohydrate and 0.8 g/kg BM protein) in a randomized crossover design. Venous blood samples, body mass, body water, and breath samples were collected, and gastrointestinal symptoms (GIS) were measured, pre- and post-exercise, and during recovery. Muscle biopsies were performed at 0 and 2 h of recovery. The following morning, participants returned to the laboratory to assess performance outcomes. In the recovery period, carbohydrate malabsorption (breath H_2_ peak: 49 *vs*. 24 ppm) occurred on MBSB compared to CM, with a trend toward greater gut discomfort. No difference in gastrointestinal integrity (i.e., I-FABP and sCD14) or immune response (i.e., circulating leukocyte trafficking, bacterially-stimulated neutrophil degranulation, and systemic inflammatory profile) markers were observed between CM and MBSB. Neither trial achieved a positive rate of muscle glycogen resynthesis [−25.8 (35.5) mmol/kg dw/h]. Both trials increased phosphorylation of intramuscular signaling proteins. Greater fluid retention (total body water: 86.9 *vs*. 81.9%) occurred on MBSB compared to CM. Performance outcomes did not differ between trials. The greater nutrient composition of MBSB induced greater gastrointestinal functional disturbance, did not prevent the post-exercise reduction in neutrophil function, and did not support greater overall acute recovery.

## Introduction

Sports focused food and supplement products are commonly used by athletes within elite and recreational level endurance sporting communities (1). Current trends include the popularity of dairy based “whole foods” and “specially formulated supplement” beverages to promote exercise recovery. Flavored whole food dairy milk beverages are considered a “gold standard” exercise recovery beverage, due to the close alignment of the quality and quantity of their nutrient composition with current exercise recovery nutrition guidelines and recommendations (2, 3). However, reconstituted dairy milk based supplement beverages are favored by athletes, but generally contain higher energy and nutrient (e.g., carbohydrate and protein) content per volume due to their concentrated formulation (4). According to current exercise recovery nutrition guidelines and recommendations, the immediate post-exercise intake of 1.0–1.2 g/kg body mass (BM) of carbohydrate and 0.2–0.4 g/kg BM of protein will support muscle glycogen resynthesis, muscle protein synthesis. In addition, these quantities of post-exercise carbohydrate and protein intake have been shown to support immune status in response to immunodepressive exercise (e.g., 2 h running at ≥70% *V*O_2max_) (5–8). For example, the consumption of a recovery beverage (i.e., supplementation or whole food source) providing 1.2 g/kg BM of carbohydrate with or without 0.4 g/kg BM of protein, immediately after exercise cessation, has consistently been shown to prevent the post-exercise reduction in bacterial endotoxin challenged (i.e., *E.coli* lipopolysaccharide) neutrophil function *in-vitro* (5, 7, 9). Unlike other immune functional responses (e.g., *in-vitro* lymphocyte proliferation and *in-vivo* delay type hypersensitivity), bacterially-stimulated neutrophil degranulation (i.e., elastase concentration)has consistently been shown to response to recovery nutrition (5, 7, 9); and is potentially a fundamental immune function to assist recovery processes (e.g., systemic bacterial and bacterial endotoxin clearance, and soft tissue repair), and required to re-establish gastrointestinal-associated lymphoid structure and function that needed to support the bioavailability of ingested nutrients after exercise (10, 11). Fluid intake equivalent to 125% to 150% of the exercise-induced BM loss is recommended for restoration of hydration status to pre-exercise levels, with sodium-containing fluids proposed to drive thirst and promote extracellular fluid retention (12). In line with these recommendations, flavored dairy milk beverages contain carbohydrate and protein in an approximate ratio of 3 to 4:1, and a naturally high sodium content (50–100 mg/100 ml) (13). A recent systematic literature review found that dairy based exercise recovery beverages may enhance recovery outcomes including muscle glycogen resynthesis, muscle protein synthesis, rehydration, and subsequent endurance exercise performance when compared to non-dairy recovery beverages (4). However, this review did not distinguish the nutritional composition (i.e., nutrient quantity and quality) of beverages, nor differentiate outcomes between the types of dairy products (e.g., dairy milk, dairy based sports beverages, and other variants); and the gastrointestinal tolerance (e.g., regulation of nutrient bioavailability) in response to gastrointestinal perturbing exercise (i.e., exercise-induced gastrointestinal syndrome) (10, 14).

Commercially, analogous of dairy based exercise recovery beverages exhibit a wide variability of nutritional compositions, sensory profiles, and price (13). For example, standard dairy milk, flavored dairy milk, and dairy milk based supplementation (e.g., casein and/or whey mixtures) beverages, respectively, increase in nutritional density (e.g., 0.6–2.4 g kg BM of carbohydrate and 0.2–0.8 g/kg BM protein) at an isovolumetric dose; but are equally promoted to act as exercise recovery agent in professional practice, despite nutritional availability may be under or over general recovery nutrition guideline and recommendations (3). Variability in recovery beverage processing (e.g., carbohydrate and protein type, fat and electrolyte content- quality) and nutritive composition (e.g., load- quantity) alters the gastrointestinal transit, digestibility and assimilation of nutrients in whole dairy products compared to isolated nutrient supplements (e.g., whey protein or glucose powders) (15). To date, comparative studies on milk beverages for exercise recovery are generally limited to markers of hydration, gastrointestinal symptoms (GIS), and performance (16–19). These studies provide some evidence of enhanced fluid retention with a reconstituted dairy based exercise recovery beverage, compared to soy and regular dairy milks (i.e., bovine source) (16). No other differences in fluid retention, GIS, or performance have been observed between differing dairy based exercise recovery beverages; while, other integrated markers of recovery optimisation (i.e., gastrointestinal integrity and function muscle, immune function and status, glycogen resynthesis, and muscle protein synthesis) are yet to be comprehensively explored.

Given the prevalent use of exercise recovery beverages amongst athletes, there has been encouragement by sport and exercise practitioners (e.g., sport or performance dietitians and nutritionists) for well-controlled experimental trials to evaluate the efficacy of these products and develop evidenced based protocols for use (20). Flavored dairy milk beverages and reconstituted dairy milk based supplement beverages are the most commonly consumed beverages amongst athletic populations in the post-exercise recovery period, and appear to meet or over-provide post-exercise nutritional requirements (4, 13). However, to date, the impact of this nutritional discrepancy provision has not been explore in regards to gastrointestinal and immune status, and subsequent impact on more traditional markers of exercise recovery nutrition (i.e., refuel, repair, and rehydration). With this in mind, the current study aimed to determine the effects of flavored dairy milk recovery beverages of different energy and nutrient densities (i.e., standard *vs*. high nutritional content), but same intake volume, on markers of gastrointestinal and immune status, and subsequent recovery optimisation markers (i.e., muscle glycogen resynthesis, protein synthesis expression, and hydration). It was hypothesized that the higher energy and nutrient density of a dairy milk based supplement beverage will result in greater muscle glycogen resynthesis, muscle protein synthesis, fluid retention, and enhanced immune functional responses, compared with a standard nutrient content of the whole food dairy milk recovery beverage. In addition, the greater energy and nutrient density of the reconstituted dairy milk based supplement beverage will result in greater ratings of feeding intolerance and GIS, compared with a standard nutrient content of the whole food dairy milk recovery beverage.

## Methods

### Participants

Nine amateur recreationally and competitively trained endurance athletes (*n* = 7 male, *n* = 2 female) [mean (standard deviation (SD)]: age 28 (4) years, nude BM 73.3 (14.1) kg, height 1.76 (0.12) m,% body fat 13.6 (7.4)%, *V*O_2max_ 53 (3) ml/kg BM/min, weekly training volume [447 (260) min, and training/competition modality: endurance running, ultra-endurance running, triathlon] volunteered to participate in the study. Participants responded to poster advertisements displayed on social media and in relevant sports clubs in Melbourne, Australia. All participants gave written informed consent. The current study was part of a larger project for which the protocol was prospectively registered with ANZCTR (reference number 375090) and participants were randomly allocated to respective experimental procedures using a computer sequence generator. The study received approval from the local ethics committee (MUHREC: 12799) and conformed to the Helsinki Declaration for Human Research Ethics. All participants confirmed the absence of illness, injury, or disease, including gastrointestinal infections, diseases and/or disorders. Individuals were excluded if they reported the consumption of potential dietary modifiers of gastrointestinal integrity, were adhering to gastrointestinal-focused dietary regimens within the previous 3 months, or had consumed non-steroidal anti-inflammatory medications, antibiotic or stool altering medications within 1 month before the experimental protocol, or if they failed to achieve a *V*O_2max_ > 50 ml/kg BM/min.

### Preliminary Measures

Baseline measurements were recorded 1–3 weeks prior to the first experimental trial. Height (stadiometer, Holtain Limited, Crosswell, Crymych, United Kingdom) and BM (Seca 515 MBCA, Seca Group, Hamburg, Germany) were recorded. *V*O_2max_ (Vmax Encore Metabolic Cart, Carefusion, San Diego, California, US) was estimated using a continuous incremental exercise test to volitional exhaustion on a motorized treadmill (Forma Run 500, Technogym, Seattle, Washington, US), as previously reported (5). In short, the exercise test began with a treadmill speed of 6 km/h and 1% inclination. Speed was increased by 2 km/h every 3 min until reaching 16 km/h at which point inclination was increased by 2.5% every 3 min until the participant reached volitional exhaustion and criteria for attaining *V*O_2max_ (i.e., heart rate, rating of perceived exertion, and respiratory exchange ratio). From the *V*O_2_-work rate relationship, the treadmill speed at 1% inclination corresponding to 50% [6.6 (0.6) km/h], 60% [7.8 (1.0) km/h], 70% [9.4 (1.1) km/h], and 80% [11.6 (1.3) km/h] *V*O_2max_ was extrapolated and verified.

### Experimental Protocol

Participants were required to consume a standardized diet in accordance with current nutrition guidelines for endurance athletes (3), and low in fermentable carbohydrate (FODMAPs) during the 24 h prior to, and throughout the experimental trials [3,018 (1,754) ml/day water, 10.6 (3.0) MJ/day, 101 (32) g protein/day, 64 (33) g fat/day, 364 (67) g carbohydrates/day, and 44 (10) g/day fiber]. Diets were designed to provide <2 g FODMAPs per meal using a Monash University designed FODMAP specific database (FoodWorks Professional 7, Xyris, Brisbane, Australia) (21). Meal provisions were stratified according to BM, such that participants with greater body mass were provided with additional meal servings and (or) snacks. Participants were asked to refrain from consuming alcohol, and performing strenuous exercise during the 48 h before each experimental trial, and refrain from consuming caffeinated beverages during the 24 h before each experimental trial. Compliance to these instructions was checked via the completion of a food and exercise diary.

In a randomized order, participants completed two experimental trials separated with at least a 5 day washout period, to accommodate the participants' availability, and providing sufficient time to recovery primary and secondary variables to baseline (4). In the case of the female participants, resting estrogen levels (DKO003/RUO; DiaMetra, Italy) were measured for verification, and did not differ between trials [6.0 (3.0) pg/ml]. Participants reported to the laboratory at 0800 h after consuming the standardized low FODMAP mixed carbohydrate breakfast [245 (130) ml water, 2.9 (0.9) MJ, 25 (5) g protein, 22 (6) g fat, and 95 (34) g carbohydrates, and 11 (4) g fiber] (21). Before commencing exercise, participants were asked to void, and pre-exercise nude BM and total body water (TBW) (Seca 515 mBCA, Seca Group, Hamburg, Germany) were recorded. Participants inserted a thermocouple 12 cm beyond the external anal sphincter to record pre- and post-exercise rectal temperature (Precision Temperature 4,600 Thermometer, Alpha Technics, California, USA). Participants provided a breath sample into a 250 ml breath collection bag (Wagner Analysen Technick, Bremen, Germany), and completed an exercise specific modified visual analog scale GIS assessment tool (21). Blood was collected by venepuncture from an antecubital vein into three separate vacutainers (6 ml 1.5 IU/ml lithium heparin, 4 ml 1.6 mg/ml K_3_EDTA, and 5 ml SST; BD, Oxford, UK).

**Figure 1:**
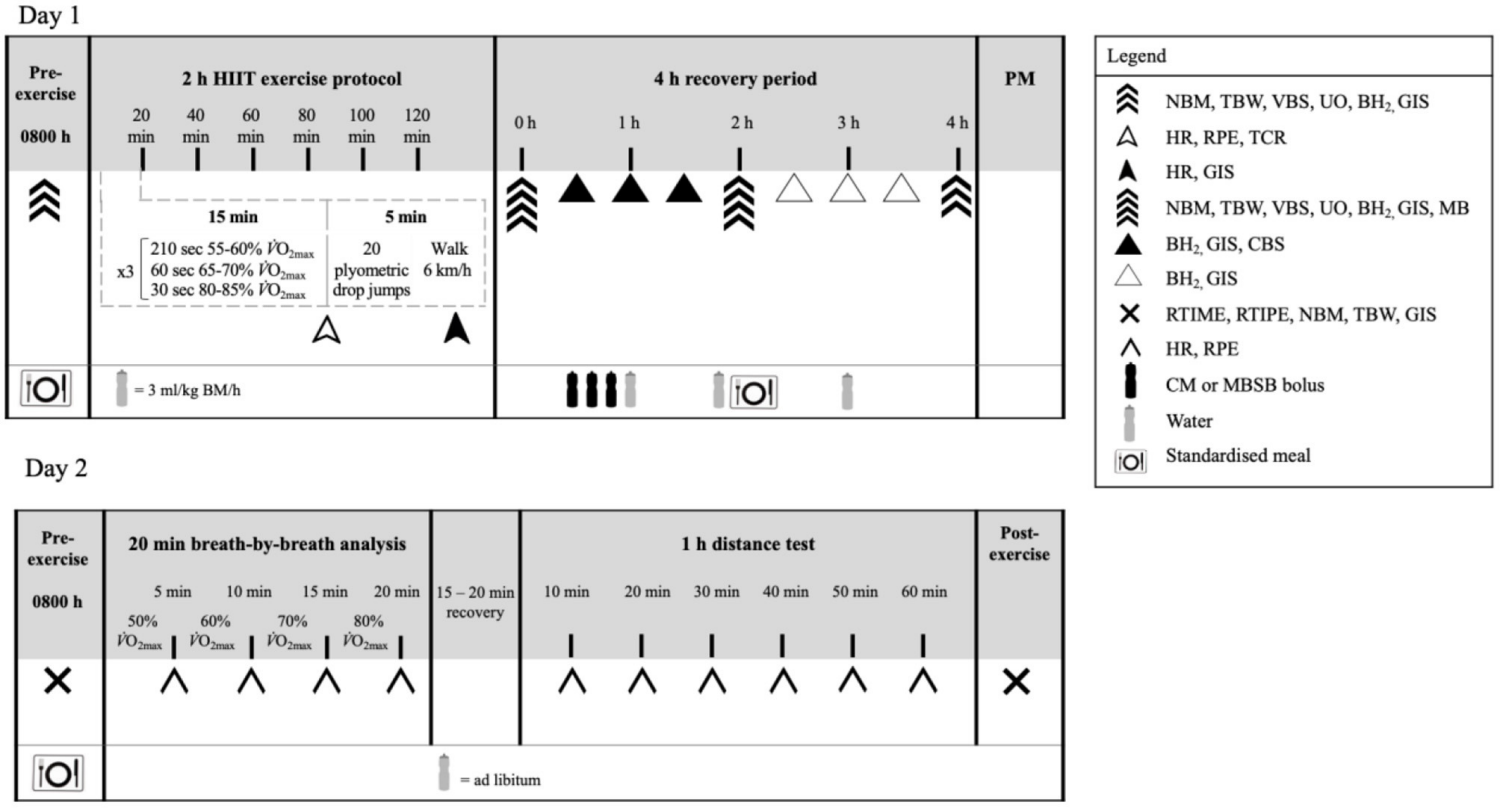
Schematic illustration of the experimental design. NBM, nude body mass; TBW, total body water; VBS, venous blood sampling; UO, urine output and osmolality; BH_2_, breath hydrogen; GIS, gastrointestinal symptoms; Tre, rectal temperature; HR, heart rate; TCR, thermal comfort rating; RPE, rating of perceived exertion; MB, muscle biopsy; CBS, capillary blood sampling; RTIME, readiness to invest mental effort; RTIPE, readiness to invest physical effort; RER, respiratory exchange ratio; CM, chocolate flavored dairy milk; MBSB, milk-based sports beverage; BM, body mass.

The exercise protocol consisted of a 2 h (initiated at 0900 h) high intensity interval exercise (HIIT) session in 23.4 (0.7)°C ambient temperature and 42 (8)% relative humidity, as described in Figure 1. The protocol was designed to provide sufficient exercise stress to perturb key markers of recovery (4, 6, 7, 22). During exercise, participants were provided with water equivalent to 3 ml/kg BM/h (5, 9). Heart rate (Polar Electro, Kempele, Finland), rating of perceived exertion, and thermal comfort rating were measured at the 15 min mark of each 20 min cycle. Recovered heart rate and GIS were measured during the final 30 s of the 20 min cycle. Immediately post-exercise, nude BM and rectal temperature were recorded. The recovery period commenced 30 min after the end of the exercise protocol to prepare for muscle biopsy sampling. Participants rested in a supine position in a sterile phlebotomy room for venous blood sampling followed by the first muscle biopsy thereafter. Muscle biopsy samples were taken 0 h and 2 h into the recovery period. TBW was measured immediately after muscle biopsy sampling. Blood samples, nude BM and TBW were collected again at 2 and 4 h of recovery. Breath samples were collected and GIS recorded every 30 min throughout the recovery period. Total urine output was collected throughout the total recovery period. Weight of urine output was recorded at 2 h and 4 h of recovery. After sampling at 2 h of recovery, participants received a standardized recovery meal [415 (103) ml water, 2.8 (0.7) MJ, 31 (8) g protein, 4 (2) g fat, and 137 (32) g carbohydrates, and 9 (2) g fiber], and were instructed to consume as much as tolerable. The total weight of the meal consumed was recorded. In addition, participants consumed a standardized low FODMAP evening meal after leaving the laboratory [818 (860) ml water, 2.8 (1.3) MJ, 31 (21) g protein, 14 (12) g fat, and 95 (46) g carbohydrates, and 18 (7) g fiber].

Participants were informed and advised to follow standardized nocturnal habits, previously assessed and reported as a control variable to current physiological and performance markers measured in the current study (23–27). This included sleeping from ~2,100–2,200 to 0600 h (~8–9 h sleep duration). However, objective data of sleep quantity and quality was not measured on this occasion. The following morning, participants returned to the laboratory (0800 h) to assess psychophysiological parameters and exercise performance (~0900 h). Due to unexpected unavailability, 1 participant did not return for the 2nd day of testing. A standardized low FODMAP mixed carbohydrate breakfast [254 (319) ml water, 2.4 (1.3) MJ, 21 (10) g protein, 19 (9) g fat, and 77 (44) g carbohydrates, and 9 (5) g fiber] was consumed at 0700 h. Nude BM, TBW and GIS were recorded on arrival and again after the performance test. Before and after the performance test, participants completed measures of readiness to invest mental and physical effort, rated from 0 to 10 (28). Participants completed a 20 min running exercise bout to measure oxygen uptake and oxidation rates at four submaximal exercise intensities (50, 60, 70, and 80% *V*O_2max_) for 5 min each, before undertaking a 1 h performance test in 23.0 (1.3) °C ambient temperature and 46 (9)% relative humidity, using methods as previously reported (14, 27, 29). Participants were instructed to run the maximal distance they are capable of running in 1 h, with the incline set at 1%. During the distance test participants only had information about elapsed time. Total distance, heart rate, rate of perceived exertion, and water intake (provided *ad-libitum*) were recorded every 10 min.

### Muscle Biopsy Procedure

Muscle biopsies were performed using a modified 5 mm Bergstrom biopsy needle. Samples were obtained from the *vastus lateralis* of the ipsilateral leg for the first trial, and contralateral leg for the second. The skin of the lateral aspect of the mid-thigh was washed well (10% Povidone – Iodine solution) then 2–3 ml of local anesthetic (lidocaine 1%) was infiltrated subcutaneously over *vastus lateralis* to anesthetize the skin and superficial fascia. After the anesthetic had taken effect, two 5 mm stab incisions ~15 mm apart were made through skin and fascia, with one incision made for each muscle biopsy sample. Samples were then extracted, immediately submerged in liquid nitrogen, and stored at −80°C prior to further analysis.

### Post-exercise Recovery Beverages

In a randomized, repeated measures design, participants were provided with [1] commercially available chocolate flavored dairy milk (CM) or [2] commercially available chocolate flavored dairy milk based supplement beverage (MBSB). The commercially available dairy milk and dairy milk based supplement beverages were kept confidential to comply with product anonymity ethical procedures. For practical relevance, and to primarily control variables from a gastrointestinal and immune recovery perspective, beverages were matched for volume (4, 7, 14). The beverages were prepared by a third party researcher, and served in opaque bottles, at ~7°C beverage temperature (30), in 3 equal boluses every 10 min, beginning 30 min into the recovery period. The volume of the beverage was calculated to provide 1.2 g/kg BM of carbohydrate and 0.4 g/kg BM of protein on CM [2,715 (514) kJ, 30 (6) g protein, 17 (3) g fat, and 92 (17) g carbohydrate]. The MBSB was prepared by dissolving 30 g dry powder in 100 ml water in accordance with manufacturers' instructions, and volume matched to the CM trial [4,029 (763) kJ, 63 (12) g protein, 2 (0) g fat, and 170 (32) g carbohydrates]. Additional water calculated to provide a total fluid intake of 35 ml/kg BM was provided at hourly intervals. Participants were instructed to drink as much as tolerable. Total fluid intake was recorded hourly. The percentage of ingested fluid retained was calculated from the difference between ingested fluid and urine output, as a fraction of total fluid intake (31).

### Sample Analysis

Blood glucose concentration, hemoglobin and total and differential leukocyte counts, which included neutrophils, lymphocytes, and monocytes, were determined by HemoCue system (Glucose 201+, Hb201, and WBC DIFF, HemoCue AB, Ängelholm, Sweden) in duplicate from heparin whole blood samples. Coefficient of variation (CV) for blood glucose concentration, hemogobin, and leukocyte counts were 5.3, 1.8, and 13.4%, respectively. Hematocrit was determined by capillary method in triplicate from heparin whole blood samples and using a microhaematocrit reader (CV: 0.5%) (ThermoFisher Scientific). Hemoglobin and hematocrit values were used to estimate changes in plasma volume (P_V_) relative to baseline, and used to correct plasma variables. To determine the blood glucose response to the recovery beverage, immediately before and every 30 min thereafter for 90 min, blood glucose concentration was measured in duplicate using a handheld system from capillary blood samples (CV: 3.7%) (Accu-Chek Proforma, Roche Diagnostics, Indianapolis, Indiana, USA). *In-vitro* bacterially-stimulated elastase release was determined using previously described methods (6). The remaining whole blood in the heparin and K_3_EDTA vacutainers were centrifuged at 4,000 rpm (1,500 g) for 10 min within 15 min of sample collection. The whole blood collected in the SST serum tube was allowed to clot for 1 h in ~4°C prior to centrifuging at 4,000 rpm (1,500 g) for 10 min. 2 × 50 μl of heparin plasma was used to determine plasma osmolality (P_Osmol_), in duplicate (CV: 2.5%), by freezepoint osmometry (Osmomat 030, Gonotec, Berlin, Germany). The remaining heparin, K_3_EDTA and serum plasma was aspirated into the appropriate 1.5 ml micro-storage tubes and frozen at −80°C until analysis. Circulating concentrations of insulin (DKO076; DiaMetra, ItalyRE53171; IBL International, Hamburg, Germany), cortisol (DKO001; DiaMetra, ItalyRE52061; IBL International, Hamburg, Germany), aldosterone (Demeditec Diagnostics GmbH, Kiel, Germany), PMN elastase (BMS269; Affymetrix EBioscience, Vienna, Austria), intestinal fatty acid-binding protein (I-FABP) (HK406; Hycult Biotech, Uden, The Netherlands), and sCD14 (HK320; Hycult Biotech), were determined by ELISA. Additionally, systemic cytokine profile (including plasma interleukin (IL)-6, IL-1β, tumor necrosis factor (TNF)-α, IL-8, IL-10, and IL-1 receptor antagonist (ra) concentrations) (HCYTMAG-60K, EMD Millipore, Darmstadt, Germany) were determined by multiplex system. All variables were analyzed as per manufacturer's instructions on the same day, with standards and controls on each plate, and each participant assayed on the same plate. The CVs for ELISAs were ≤6.1% and for cytokine profile multiplex was 16.0%. Breath samples (20 ml) were analyzed in duplicate (CV: 2.1%) for hydrogen (H_2_) content using a gas-sensitive analyser (Breathtracker Digital Microlyzer, Quintron, Milwaukee, Wisconsin, US). Plasma sodium, potassium and calcium concentrations were determined using ion selective electrodes (Cobas c analyser, Roche Diagnostics, Risch-Rotkreuz, Switzerland) and analyzed by local pathology services (Cabrini Pathology, Malvern, Victoria, Australia).

### Western Blot Analysis

Approximately 30 mg of skeletal muscle was solubilized in radioimmunoprecipitation buffer (Millipore, Bayswater, Victoria, Australia) with 1 μL/ml protease inhibitor cocktail (Sigma-Aldrich, Castle Hill, New South Wales, Australia) and 10 μl/ml Halt Phosphatase Inhibitor Single-Use Cocktail (Thermo Scientific, Australia, North Ryde, New South Wales, Australia). The concentration of protein per sample was determined by the bicinchoninic acid assay (BCA Protein Assay Kit#23225, Thermo Scientific). Twenty microgram of skeletal muscle protein lysate was loaded onto into either Bio-Rad precast Criterion TGX Stain-Free 4 to 12% gels (Bio-Rad, Gladesville, New South Wales, Australia). SDS-PAGE was conducted following manufacturer's instructions. Protein was then transferred to PVDF membranes and blocked for 1 h in 5% bovine serum albumin (BSA) solution in Tris-buffered saline-Tween, (pH 7.6, 20 mmol/L Tris and 150 mmol/L NaCl, 0.1% Tween) (TBST) at room temperature. Membranes were then incubated in primary antibodies diluted in 5% BSA/TBST overnight at 4°C. Following washing in TBST, membranes were incubated for 1 h with fluorescent secondary antibodies (phospho-mammalian target of rapamycin ${\left(\text{mTOR}\right)}_{,}^{\text{Ser}2448}$ phospho-protein kinase B (Akt)^Ser473^, phospho-ribosomal protein S6 (rpS6)^Ser235/236^, and phosphor-glycogen synthase kinase 3β (GSK-3β)^Ser9^) (Anti-Rabbit IgG (H+L) DylightTM 800 Conjugate; Anti-mouse IgG (H+L) DylightTM 680 Conjugate) (Cell Signaling Technologies®, Danvers, Massachusetts, USA) diluted 1:10,000 in TBST. Following 2 further washes in TBST and 1 wash in phosphate buffered saline (PBS) membranes were scanned using the LiCOR® Odyssey CLx® Imaging System (Millennium Science, Mulgrave, Victoria, Australia). All targets were normalized to total protein using either the Bio-Rad stain-free system.

### Muscle Glycogen Analysis

One fraction of muscle sample (20–25 mg wet weight) was freeze-dried, after which collagen, blood and other non-muscle material were removed from the muscle fibers. Samples were then pulverized and powdered. Samples were extracted with 0.5 M perchloric acid (HClO_4_) containing 1 nM EDTA and neutralized using 2.2 M KHCO_3_. Adenosine triphosphate, phosphocreatine, and creatine was determined from the supernatant by enzymatic spectrophotometric assays (32, 33). Muscle glycogen content was determined from 2 aliquots of freeze-dried muscle (2–3 mg) as previously reported (32).

### Statistical Analysis

Confirmation of adequate statistical power was determined *a priori* from the applied statistical test, mean, standard deviation, and effect size on markers of [1] gastrointestinal integrity (i.e., plasma I-FABP), function (i.e., breath hydrogen), and GIS (6, 14, 22, 34); [2] circulating leukocyte, endotoxin and cytokine profiles (5–7, 9); [3] total body water, plasma osmolality, plasma volume change (6, 7); [4] rate of skeletal muscle glycogen resynthesis (35, 36); [5] phosphorylation of intramuscular signaling proteins (37–39); and [6] performance (27, 29). Using a standard alpha (0.05) and beta value (0.80), the current participant sample size, within a repeated measures cross-over design, is estimated to provide adequate statistical power (power* 0.80–0.99) for detecting significant between- (trial) and within- (time) group differences (G*Power 3.1, Kiel, Germany). Data in the text and tables are presented as mean (SD) for descriptive method, and mean and 95% confidence interval (CI) for primary variable, as indicated. For clarity, data in figures are presented as mean and standard error of the mean (SEM), and/or mean and individual responses, as indicated. Systemic inflammatory cytokine responses are presented as raw values and systemic inflammatory response profile (SIR-profile), as previously reported (40). Both male and female participants with full data sets within each specific variable were used in the data analysis, which is in accordance previously literature demonstrating similar variable outcomes between biological sexes (30, 31, 41). There were no outliers for female participant data points for any of the primary and secondary outcome measures. All data were checked for normal distribution (Shapiro-Wilks) prior to comparative analysis. Variables with singular data points were examined using paired sample *t*-tests, or non-parametric Wilcoxon signed-rank test, when appropriate. Variables with multiple data points were examined using a two-way repeated-measures ANOVA. Assumptions of homogeneity and sphericity were checked, and when appropriate adjustments to the degrees of freedom were made using the Greenhouse-Geisser correction method. Main effects were analyzed by Tukey's *post hoc* HSD. Statistics were analyzed using SPSS statistical software (V.26.0, Chicago, Illinois, USA) with significance accepted at *P* ≤ 0.05.

## Results

### Physiological Strain

A main effect of time (MEOTime) was observed for recovered heart rate [overall mean and 95% CI: 129 (125–132) bpm; *P* = 0.006] and peak [163 (160–166) bpm; *P* = 0.080], rate of perceived exertion [13 (12, 13); *P* = 0.001], TCR [9 (9); *P* < 0.001] and core body temperature (*P* < 0.001), characterized by an increased physiological strain as the exercise stress progressed. A MEOTime occurred for plasma cortisol concentrations, such that values were significantly lower 4 h into recovery compared to the beginning of the recovery period (*P* < 0.05), associated with normal daily circadian variation (Table 1).

**Table 1:** Change in gastrointestinal and immune biomarkers in response to 2 h HIIT exercise (between 60 and 80% *V*O_2max_) in temperate ambient conditions and after consumption of a whole food dairy milk beverage (CM) and a reconstituted milk based supplement beverage (MBSB).

	**CM**				**MBSB**			
	**Pre-exercise**	**0 h recovery**	**2 h recovery**	**4 h recovery**	**Pre-exercise**	**0 h recovery**	**2 h recovery**	**4 h recovery**
Cortisol (nmol/L)	705 (620–790)	883 (570–1,096)	546 (460–632)	367^†^ (265–469)	641 (542–740)	870 (634–1,106)	646 (509–783)	420^†^ (210–630)
I-FABP (pg/ml)	545 (382–707)	1,793^§^ (714–2,873)	—	—	683 (392–975)	1,360^§^ (973–1,748)	—	—
sCD14 (μg/ml)	2.37 (2.21–2.52)	2.29 (2.12–2.46)	—	—	2.40 (2.25–2.55)	2.43 (2.18–2.68)	—	—
IL-1β (pg/ml)	1.4 (0.5–2.3)	1.2 (0.3–2.0)	1.7 (0.8–2.6)	1.3 (0.3–2.2)	1.0 (0.3–1.7)	1.1 (0.4–1.8)	1.3 (0.3–2.2)	2.0^##^ (0.7–3.3)
TNF-α (pg/ml)	6.9 (5.3–8.5)	7.0 (5.3–8.7)	7.8 (5.6–10.0)	5.9 (4.4–7.5)	6.5 (4.8–8.2)	7.3 (5.5–9.1)	6.7 (4.8–8.6)	6.8 (4.5–9.0)
IL-6 (pg/ml)	18.4 (<0.1^c^-38.3)	15.6 (<0.1^c^-31.9)	19.6 (<0.1^c^-41.8)	17.3 (<0.1^c^-36.0)	14.7 (<0.1^c^-29.2)	16.0 (<0.1^c^-31.5)	16.1 (<0.1^c^-34.1)	20.8 (<0.1^c^-46.1)
IL-8 (pg/ml)	11.3 (0.7–21.9)	10.0 (0.8–19.3)	11.2 (0.5–21.8)	10.2 (0.1–20.4)	9.3 (1.2–17.4)	10.0 (1.8–18.2)	9.1 (0.5–17.8)	11.4 (<0.1^c^-23.9)
IL-10 (pg/ml)	8.6 (4.1–13.1)	18.2 (5.6–30.9)	9.7 (3.8–15.6)	7.6 (2.8–12.5)	7.3 (3.2–11.5)	16.5 (5.7–27.3)	7.2 (2.9–11.5)	8.1 (2.8–13.4)
IL-1rα (pg/ml)	17.7 (9.0–26.4)	22.7 (11.2–34.1)	26.7 (14.2–39.1)	23.0 (14.4–31.5)	15.9 (9.5–22.4)	22.4 (13.1–31.7)	23.5 (13.9–33.0)	27.2 (10.6–43.8)

Mean (95% CI) (n = 9). MEOTime

§ p < 0.05 vs. pre-exercise, MEOTime

† *p < 0.05 vs. post-exercise*,

## *p < 0.01 and vs. CM*,

c *under detectable lowest standard*.

### Gastrointestinal Integrity, Function and Symptoms

**Figure 2:**
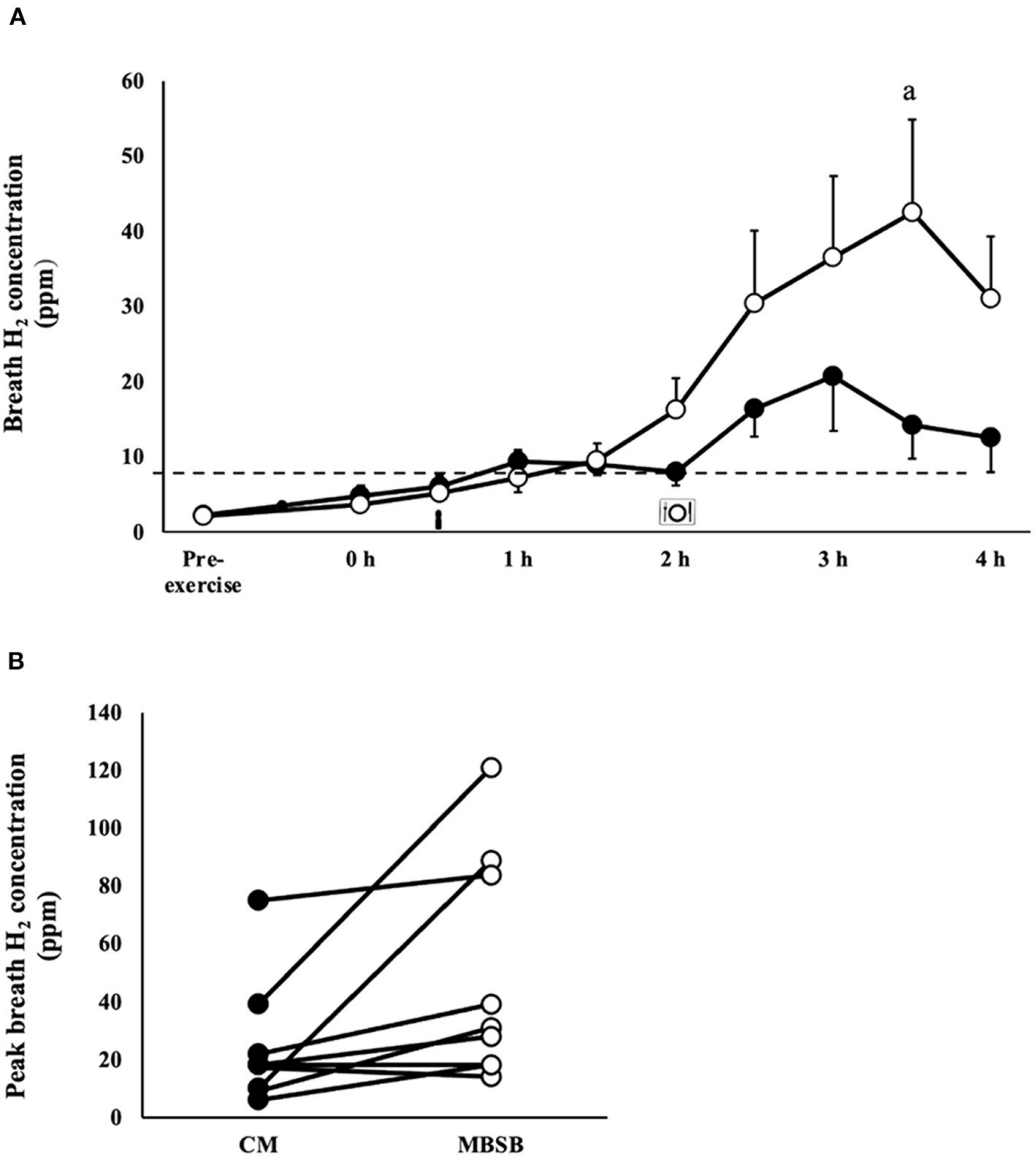
Breath hydrogen response **(A)** and individual peak breath hydrogen **(B)** after 2 h HIIT exercise in temperate ambient conditions and consumption of a whole food dairy milk (CM: ■) and a reconstituted milk based supplement (MBSB: **°**) recovery beverage. Mean ± SEM (*n* = 9): ^a^*P* < 0.05 vs. CM.

An increase in I-FABP occurred in response to the exercise stress (*P* = 0.008), with no trial differences [742 (466–1,019) pg/ml]. No significant effects or interaction were observed for plasma sCD14 concentration (Table 1). A trial*time interaction was observed for breath H_2_, with significantly greater concentrations observed on MBSB at 3.5 h into recovery (*P* < 0.05; Figure 2A). Peak breath H_2_ (*P* = 0.014) was significantly greater on MBSB [49 (24–74) ppm] than CM [24 (10–38) ppm]. Both trials reached peak breath H_2_ of clinical significance (i.e., >10 ppm) between 3 and 3.5 h into recovery (Figure 2B). A corresponding MEOTime occurred for lower GIS 3.5 h (*P* < 0.01) and 4 h (*P* < 0.05) into recovery. There was a trend toward greater total gut discomfort on MBSB (*P* = 0.053). No significant main effects or interactions were observed for upper-GIS, nausea or total-GIS (Table 2).

**Table 2:** Incidence of gastrointestinal symptoms and severity of gut discomfort, total, upper-, and lower-gastrointestinal symptoms in response to 2 h HIIT exercise (between 60 and 80% *V*O_2max_) in temperate ambient conditions and after consumption of a whole food dairy milk beverage (CM) and a reconstituted milk based supplement beverage (MBSB).

	**CM**					**MBSB**				
	**Exercise**		**Recovery**			**Exercise**		**Recovery**		
	**Incidence**	**Severity**	**Incidence**	**Severity**		**Incidence**	**Severity**	**Incidence**	**Severity**	
	**%** **(severe)**		**%** **(severe)**	**Acute** **(0–2 h)**	**Total** **(0–4 h)**	**%** **(severe)**		**%** **(severe)**	**Acute** **(0–2 h)**	**Total** **(0–4 h)**
**Gut discomfort**	NA	7 (1–27)	NA	4 (1–19)	10 (2–41)	NA	6 (3–17)	NA	6 (3–16)	19 (1–48)
**Total GIS^a^**	89 (56)	11 (1–52)	89 (67)	4 (1–19)	12 (2–59)	67 (56)	8 (3–28)	89 (78)	8 (3–29)	28 (1–91)
**Upper GIS^b^**	78 (33)	5 (1–19)	56 (22)	3 (1–19)	5 (1–32)	56 (22)	3 (2–12)	56 (33)	3 (1–21)	7 (1–39)
Belching	67 (22)	2 (1–9)	11 (0)	0 (1–1)	0 (1–1)	22 (0)	1 (1–5)	22 (11)	1 (1–5)	1 (1–7)
Heartburn	33 (22)	2 (1–10)	11 (0)	0 —	0 (3–3)	22 (22)	2 (9–12)	11 (0)	0 (1–1)	0 (2–2)
Bloating	0 (0)	0 —	33 (22)	2 (5–8)	4 (3–27)	0 (0)	0 —	44 (33)	3 (1–16)	6 (1–27)
Stomach pain	11 (11)	1 (7–7)	11 (11)	0 —	1 (5–5)	11 (0)	0 (3–3)	11 (11)	0 —	1 (5–5)
Urge to regurgitate	0 (0)	0 —	0 (0)	0 —	0 —	0 (0)	0 —	0 (0)	0 —	0 —
Regurgitation	0 (0)	0 —	0 (0)	0 —	0 —	0 (0)	0 —	0 (0)	0 —	0 —
**Lower GIS^b^**	33 (22)	1 (2–6)	67 (44)	0 (2–2)	6 (2–23)	33 (22)	2 (2–9)	67 (44)	1 (6–7)	18 (1–65)
Flatulence	22 (0)	1 (2–3)	22 (22)	0 (2–2)	1 (5–6)	22 (11)	1 (2–9)	22 (0)	0 (1–1)	0 (1–1)
Lower bloating	11 (0)	0 (2–2)	56 (22)	0 —	2 (1–6)	11 (0)	1 (3–6)	44 (33)	1 (3–6)	4 (1–15)
Urge to defecate	11 (0)	0 (3–3)	11 (0)	0 —	0 (3–3)	11 (0)	0 (4–4)	44 (33)	0 —	5 (2–25)
Intestinal pain	11 (0)	0 3–3	0 (0)	0 —	0 —	0 (0)	0 —	11 (0)	0 (3–3)	0 (3–3)
Abnormal defecation^c^	0 (0)	0 —	22 (22)	0 —	2 (10–10)	0 (0)	0 —	33 (33)	0 —	8 (10–40)
**Others**										
Nausea	11 (11)	1 (5–5)	11 (0)	0 —	0 4–4	0 (0)	0 —	0 (0)	0 —	0 —
Dizziness	44 (22)	2 (2–8)	33 (11)	1 (2–7)	1 (2–9)	33 (11)	2 (2–16)	44 (33)	3 (3–13)	3 (3–13)
Stitch^d^	11 (11)	2 (19–19)	0 (0)	0 —	0 —	11 (0)	0 (3–3)	11 (0)	0 (2–2)	0 (2–2)
Appetite	NA	11 (1–33)	NA	19 (2–44)	25 (2–68)	NA	11 (1–24)	NA	20 (12–50)	24 (12–65)
Thirst	NA	24 (1–46)	NA	7 (1–16)	24 (7–42)	NA	23 (6–35)	NA	18 (3–29)	26 (3–48)

*Values are presented as means and range of participant reporting GIS incidence (n = 9). GIS incidence during exercise and recovery are presented as percentage of total participants reporting GIS ≥1 on the mVAS. GIS severity during exercise and recovery are presented as mean summative accumulation of mVAS rating scale of measured time periods and individual range of participant reporting GIS incidence (21, 42)*.

a *Summative accumulation of upper-, lower-, and other gastrointestinal symptoms*,

b *summative accumulation of upper- or lower- gastrointestinal symptoms*,

c abnormal defecation including loose watery stools, diarrhea and blood in stools, and

d *acute transient abdominal pain. NA, not applicable. Wilcoxon signed-rank tests showed no differences between CM and MBSB for GIS. Hedge's g measurement of effect size for GIS and feeding tolerance severity between CM and MBSB was determined as >0.50 and >0.80 for medium and large effects, respectively; however, no medium or large effects size value were detected between CM and MBSB*.

### Glucose Availability & Insulin Response

**Figure 3:**
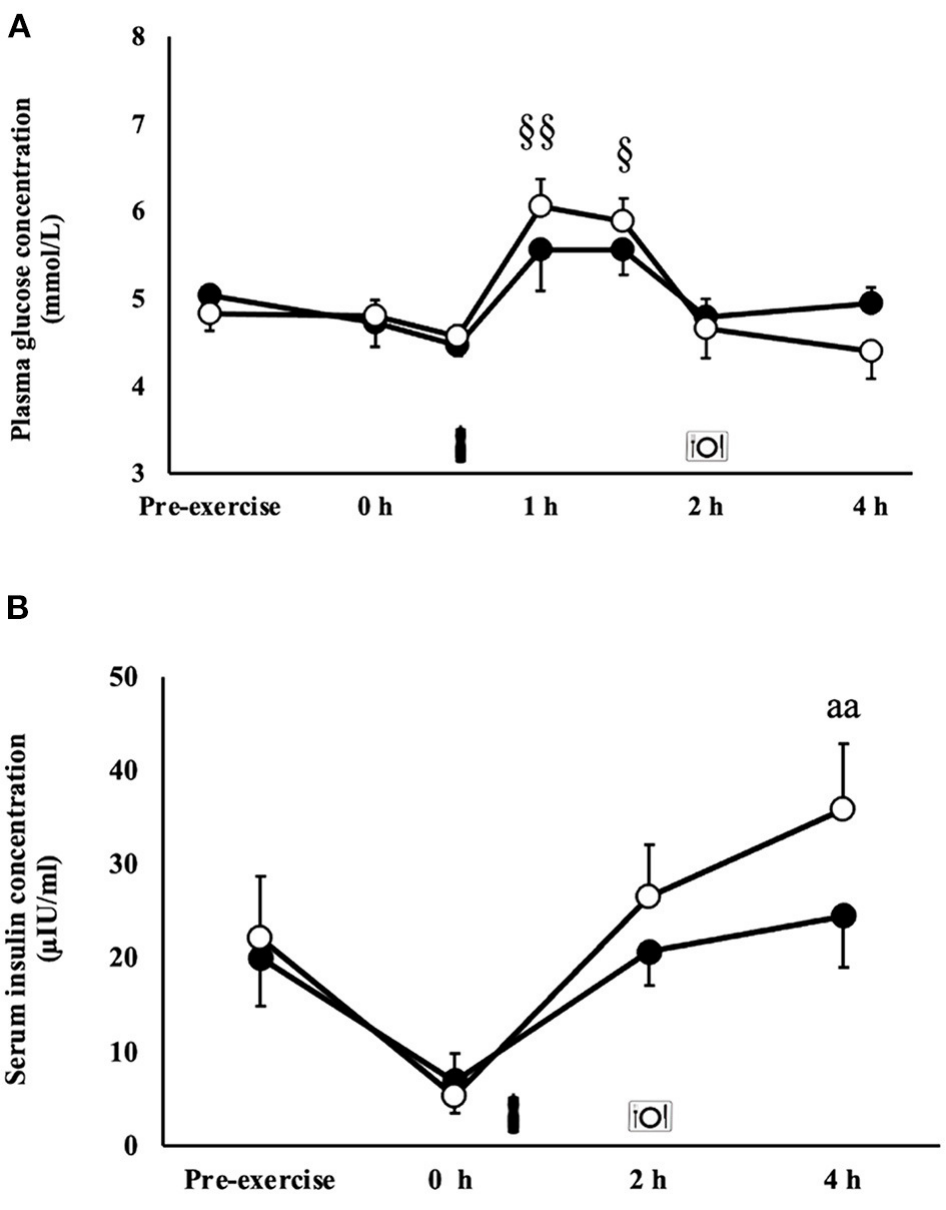
Blood glucose **(A)** and serum insulin **(B)** concentrations after 2 h HIIT exercise protocol in temperate ambient conditions and consumption of a whole food dairy milk (CM: ■) and a reconstituted milk based supplement (MBSB: **°**) recovery beverage. Mean ± SEM (*n* = 9): ^aa^*P* < 0.01 vs. CM. MEOTime ^§§^*P* < 0.01 vs. pre-exercise and ^§^*P* < 0.05 vs. pre-exercise.

There was a MEOTime for blood glucose responses, whereby blood glucose increased from the beginning to 1 h (*P* < 0.01) and 1.5 h (*P* < 0.05) into recovery (Figure 3A). A trial*time interaction occurred for serum insulin (*P* < 0.01), whereby concentrations were significantly greater 4 h into recovery on the MBSB trial [35.9 (22.2–49.5) μIU/ml] compared with CM [24.5 (13.8–35.2) μIU/ml; Figure 3B].

### Immune Responses

**Figure 4:**
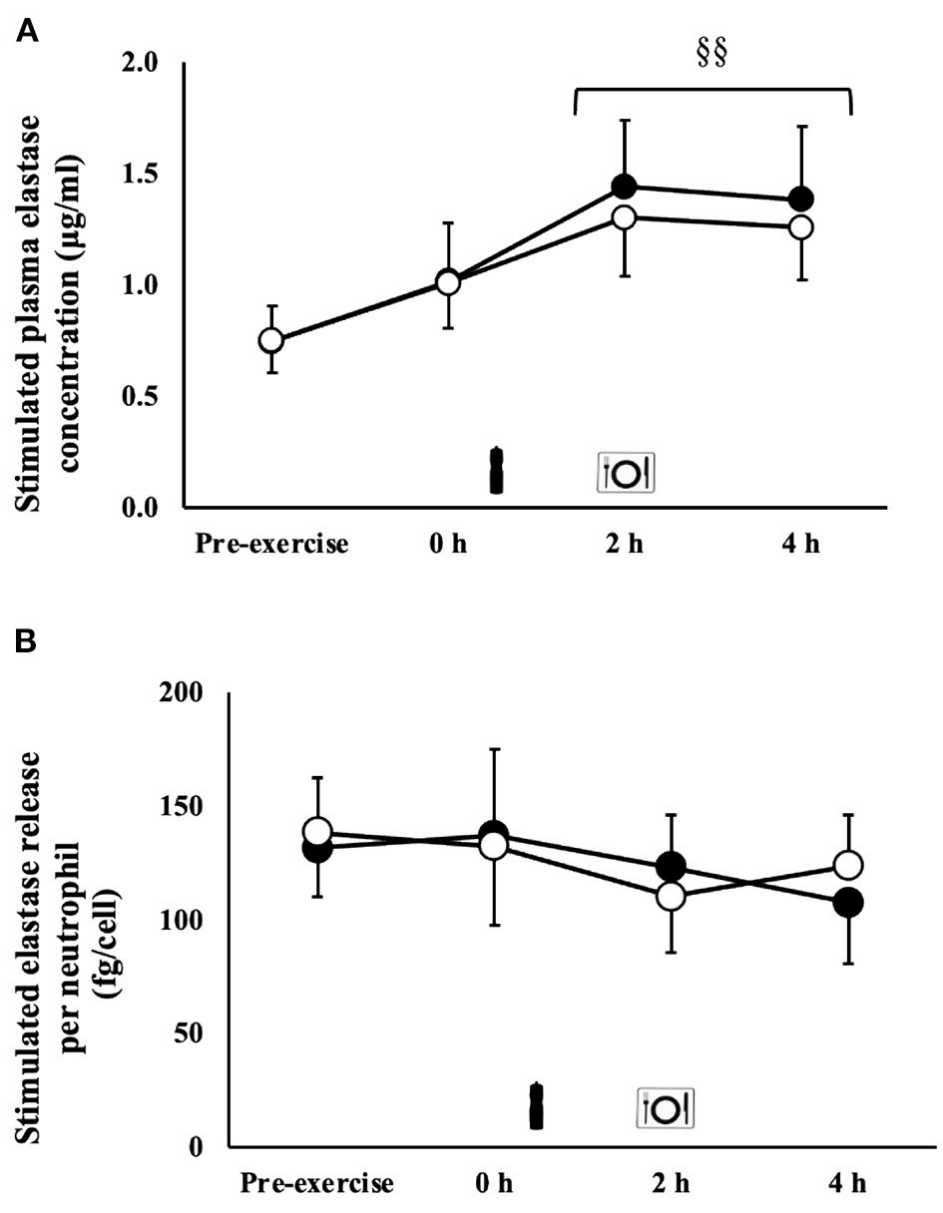
Total **(A)** and per cell **(B)** bacterially-stimulated neutrophil elastase release after 2 h HIIT exercise in temperate ambient conditions and consumption of a whole food dairy milk (CM: ■) and a reconstituted milk based supplement (MBSB: **°**) recovery beverage. Mean ± SEM (*n* = 9): MEOTime ^§§^*P* < 0.01 vs. pre-exercise.

An exercise-induced leukocytosis (MEOTime) [10.6 (3.0) × 10^9^/L; *P* = 0.001], neutrophilia [7.0 (2.3) × 10^9^/L; *P* < 0.001], lymphocytosis [3.4 (1.0) × 10^9^/L; *P* = 0.002], and monocytosis [0.7 (0.3) × 10^9^/L; *P* = 0.005] occurred in response to the exercise stress. A trial*time interaction was observed for neutrophil counts (*P* = 0.005), whereby a significantly greater neutrophil count was observed on CM [6.7 (2.6) × 10^9^/L) at 4 h of the recovery period compared with MBSB (5.3 (1.6) × 10^9^/L) (*P* < 0.01). No main effects or interaction were observed for unstimulated plasma elastase concentration [71 (47–96) ng/ml]. A MEOTime was observed for total bacterially-stimulated plasma elastase concentration (*P* = 0.001), such that values increased during recovery (Figure 4A). Bacterially-stimulated elastase release per neutrophil *in-vitro* did not differ between CM and MBSB [125 (107–144) fg/cell]. Neutrophil function decreased by 4 h of recovery during the CM (-18%) trial and 2 h of recovery during the MBSB (−20%) trial (Figure 4B), however this change was not significant.

### Systemic Inflammatory Cytokine Profile

A trial*time interaction occurred for IL-1β concentrations, whereby values were significantly higher on MBSB 4 h into recovery [2.0 (0.6–3.4) pg/ml] compared to CM [1.3 (0.2–2.3) pg/ml] (*P* < 0.01). No significant effects or interaction were observed for plasma TNF-α, IL-6, IL-8, IL-10, and IL-1ra concentrations. Exercise-induced SIR-profile [CM: 22 (12–32) arb.unit and MBSB: 38 (7 to 69) arb.unit] and recovery beverage post-prandial SIR-profile [CM: 4 (−20 to 29) arb.unit and MBSB: 6 (−26 to 38) arb.unit] did not differ between trials (Table 1).

### Muscle Glycogen Resynthesis

**Figure 5:**
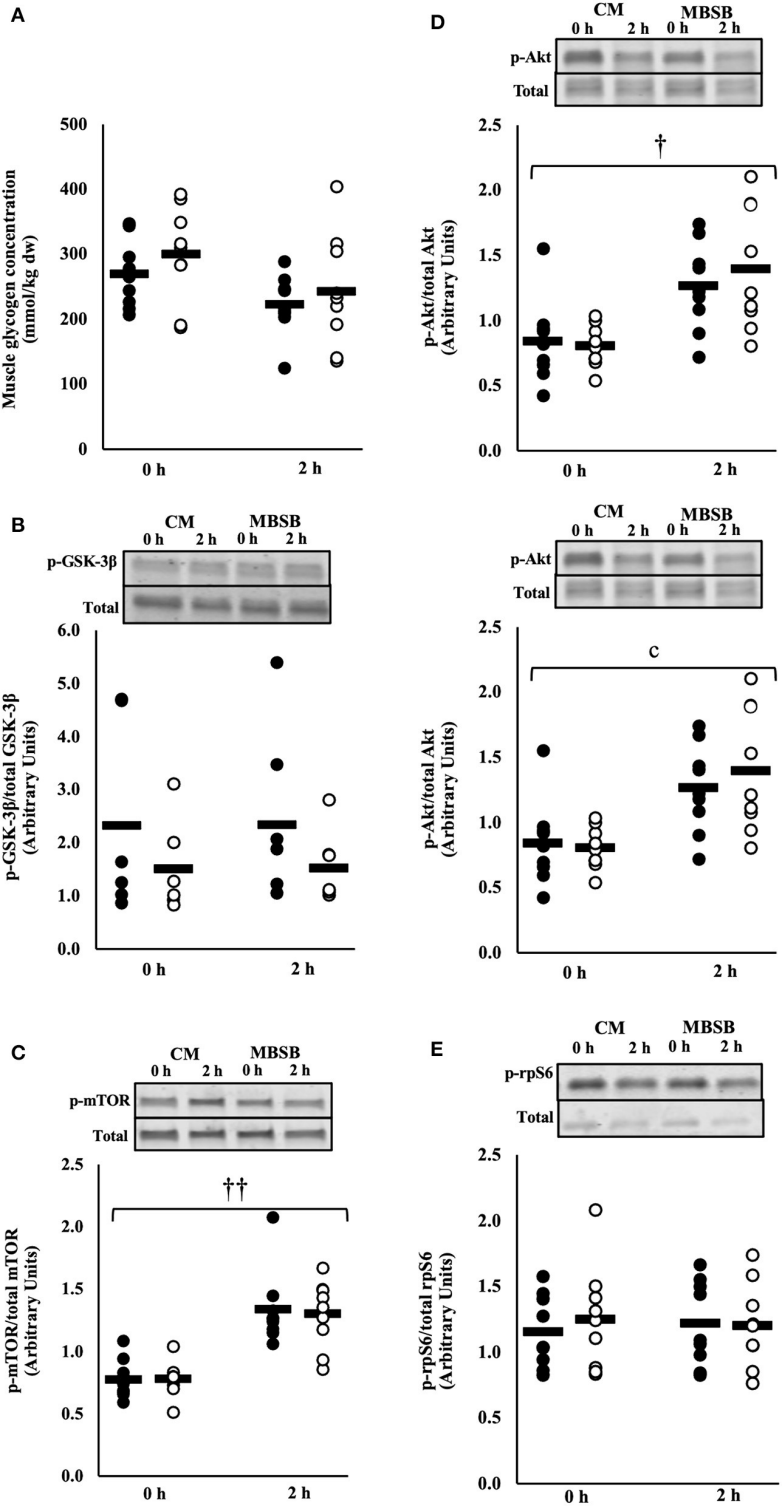
Muscle glycogen content **(A)**, phosphorylated GSK3-β to total GSK3-β **(B)**, ratio of phosphorylated mTOR to total mTOR **(C)**, phosphorylated Akt to total Akt **(D)**, and phosphorylated rpS6 to total rpS6 **(E)** after 2 h HIIT exercise in temperate ambient conditions and consumption of a whole food dairy milk (CM: ■) and a reconstituted milk based supplement (MBSB: □) recovery beverage. Mean and individual responses (*n* = 9): MEOTime ^††^*P* < 0.01 vs. 0 h recovery, ^†^*P* < 0.05 vs. 0 h recovery.

No significant main effects or interaction were observed for muscle glycogen concentration or rate of resynthesis. Likewise, no main effects or interaction occurred for the ratio of phosphorylated GSK-3β to total GSK-3β, or fold change of this ratio from the beginning to the 2 h into the recovery period (Figures 5A,B).

### Phosphorylation of Muscle Signaling Proteins

A MEOTime was observed for the ratio of phosphorylated mTOR to total mTOR (p-mTOR/total mTOR) (*P* < 0.001) and phosphorylated Akt to total Akt (p-Akt/total Akt) (*P* = 0.011), whereby phosphorylation of both proteins increased 2 h into the recovery period (Figures 5C,D). No time or trial interactions were observed for the ratio of phosphorylated rpS6 to total rpS6 (Figure 5E).

### Hydration Status

A significant difference in resting TBW was observed [CM: 61 (58–63)% *vs*. MBSB: 60 (58–63)%; *P* = 0.020], however all participants commenced within range of euhydration (i.e., >55%). Exercise-induced BM loss did not differ between CM and MBSB [1.7 (1.4–2.0)%]. A MEOTime was observed for plasma aldosterone concentrations pre- to post-exercise (*P* < 0.05). Total fluid intake during the recovery period did not differ between trials [CM: 24.6 (21.2–28.0) ml/kg BM and MBSB: 24.6 (22.9–26.4) ml/kg BM]. Greater fluid retention (CM: 81.9% *vs*. MBSB: 86.9%; *P* = 0.028) with a corresponding lower urine output [CM: 415 (264–567) ml *vs*. MBSB: 284 (190–379) ml; *P* = 0.015] was observed on MBSB. No significant main effects or interaction were observed for P_Osmol_, P_V_ change or plasma electrolyte concentrations (Table 3).

**Table 3:** Change in hydration markers in response to 2 h HIIT exercise (between 60 and 80% *V*O_2max_) in temperate ambient conditions and after consumption of a whole food dairy milk beverage (CM) and a reconstituted milk based supplement beverage (MBSB).

	**CM**				**MBSB**			
	**Pre-exercise**	**0 h recovery**	**2 h recovery**	**4 h recovery**	**Pre-exercise**	**0 h recovery**	**2 h recovery**	**4 h recovery**
Total body water (%)	60.7 (58.4–63.0)	61.6 (59.3–63.9)	60.3^†^ (58.5–62.1)	60.7^†^ (58.6–62.7)	60.2^#^ (57.9–62.6)	61.6 (59.1–64.0)	60.4^†^ (58.0–62.8)	60.7^†^ (58.5–62.9)
(L)	44.5 (39.3–49.7)	44.2 (39.1–49.4)	44.1 (38.8–49.4)	44.8 (39.5–50.1)	44.2 (39.2–49.2)	44.3 (39.1–49.5)	44.3 (39.1–49.6)	44.9 (39.7–50.0)
Extracellular (%)	24.4 (23.4–25.3)	24.2 (23.3–25.0)	24.0 (23.1–24.9)	24.1 (23.1–25.0)	24.1 (23.1–25.1)	24.1 (23.1–25.1)	23.9 (23.0–24.9)	24.0 (23.0–25.0)
(L)	17.8 (15.9–19.7)	17.3 (15.4–19.3)	17.5 (15.5–19.5)	17.7 (15.8–19.6)	17.6 (15.7–19.5)	17.3 (15.3–19.2)	17.5 (15.6–19.5)	17.7 (15.8–19.6)
Plasma osmolality (mOsmol/kg)	295 (289–301)	293 (286–300)	294 (289–299)	294 (288–300)	290 (286–295)	291 (285–297)	292 (287–298)	292 (285–298)
Δ P_V_ (%)	—	0.8 (−1.8 to 3.4)	3.5 (−2.6 to 9.5)	1.8 (−1.9 to 5.5)	—	0.9 (−3.7 to 5.5)	0.6 (−3.3 to 2.1)	1.4 (−1.8 to 4.5)
Aldosterone (nmol/L)	165 (118–212)	436^§^ (288–584)	178 (136–221)	127 (101–152)	180 (104–256)	446^§^ (306–586)	178 (116–239)	147 (124–169)
Serum sodium (mmol/L)	141 (139–143)	143 (139–147)	137 (137–155)	143 (137–149)	141 (140–142)	142 (136–149)	142 (136–149)	144 (138–150)
Serum potassium (mmol/L)	4.7 (4.3–5.0)	4.6 (4.3–4.8)	4.5 (4.2–4.7)	4.5 (4.1–4.9)	4.6 (4.2–5.1)	4.4 (4.1–4.7)	4.4 (4.2–4.6)	4.5 (4.4–4.7)
Serum calcium (mmol/L)	2.32 (2.26–2.38)	2.29 (2.22–2.36)	2.43 (2.30–2.56)	2.40 (2.28–2.51)	2.34 (2.29–2.39)	2.29 (2.17–2.41)	2.37 (2.27–2.47)	2.39 (2.30–2.47)

Mean (95% CI) (n = 9). MEOTime

§ p < 0.05 vs. pre-exercise, MEOTime

† *p < 0.05 vs. post-exercise*,

# *p < 0.05 vs. CM*.

### Psychophysiological Parameters & Subsequent Performance

Prior to the performance tests, participants reported greater readiness to invest physical effort on MBSB (*P* = 0.001). Readiness to invest physical effort decreased on both trials from pre- to post-exercise (*P* = 0.021). No main effects or interaction were observed for readiness to invest mental effort. There were no trial differences in carbohydrate and fat oxidation rates or physiological parameters at any stage of the incremental test (Table 4). There were no differences in distance covered during the 1 h running performance test [10.5 (9.7–11.2) km], and no significant differences in heart rate [171 (168–174) bpm] or rate of perceived exertion [16 (15,16)] throughout the distance test on CM and MBSB.

**Table 4:** Physiological and performance outcomes graded intensity breath-by-breath testing at 50–80% *V*O_2max_ and 1 h self-paced distance test, following the consumption of a whole food dairy milk beverage (CM) and a reconstituted milk based supplement beverage (MBSB) after 2 h HIITthe previous day.

	**CM**				**MBSB**			
	**50% *V*O_**2max**_**	**60% *V*O_**2max**_**	**70% *V*O_**2max**_**	**80% *V*O_**2max**_**	**50% *V*O_**2max**_**	**60% *V*O_**2max**_**	**70% *V*O_**2max**_**	**80% *V*O_**2max**_**
RER	0.905 (0.882–0.927)	0.936 (0.904–0.968)	0.938 (0.909–0.967)	0.965 (0.930–0.999)	0.911 (0.898–0.924)	0.928 (0.917–0.940)	0.935 (0.912–0.957)	0.963 (0.933–0.993)
*V*O_2_ (ml/kg BM/min)	24.4 (21.6–27.2)	31.7 (29.5–34.0)	37.7 (34.2–41.2)	45.3 (41.8–48.9)	24.2 (21.1–27.3)	32.2 (30.4–34.2)	38.6 (36.4–40.7)	45.2 (42.5–47.9)
Carbohydrate oxidation (g/min)	1.6 (1.2–2.0)	2.4 (1.8–2.9)	2.9 (2.2–3.5)	3.8 (3.0–4.6)	1.6 (1.3–1.9)	2.3 (2.0–2.6)	2.9 (2.4–3.3)	3.7 (3.2–4.3)
Fat oxidation (g/min)	0.3 (0.2–0.3)	0.3 (0.1–0.4)	0.3 (0.2–0.4)	0.2 (0.1–0.4)	0.3 (0.2–0.3)	0.3 (0.2–0.4)	0.3 (0.2–0.4)	0.2 (0.1–0.4)
HR (bpm)	120 (110–130)	142 (135–149)	156 (146–166)	172 (163–181)	117 (106–128)	139 (130–149)	158 (149–166)	173 (167–179)
RPE (6–20)	9 (8–10)	10 (9–12)	13 (11–14)	15 (14–17)	9 (8–10)	11 (10–11)	12 (11–13)	15 (13–16)

*Mean (95% CI) (n = 8). RER, respiratory exchange ratio; HR, heart rate; RPE, rating of perceived exertion*.

## Discussion

The current study aimed to determine the effects of flavored dairy milk based recovery beverages of different nutrition compositions, but same intake volume, on markers of gastrointestinal and immune status, and subsequent recovery optimisation markers. In accordance with our hypothesis, significantly greater carbohydrate malabsorption was observed after consumption of the MBSB, accompanied by a trend toward greater gut discomfort. However, the greater nutritional content on MBSB did not influence any immune markers during the acute recovery period. Both beverages increased phosphorylation of mTOR and Akt; however, contrary to our hypothesis, there were no differences between trials. Additionally, neither beverage was associated with measurable increases in skeletal muscle glycogen stores within 90 min of consumption. In accordance with the hypothesis, greater fluid retention was observed with the MBSB beverage, however there were no trial differences in TBW or plasma hydration markers. The observed differences in the assimilation of recovery nutrition and acute recovery outcomes did not result in differences in substrate oxidation or performance the following day, suggesting overall dietary intake over the post-exercise period to the following day supersedes any impact of recovery optimisation markers in response to an acute exercise recovery beverage. From a professional practice perspective, considering the current study is the first to assess the impact of dairy milk recovery beverages with differing nutritional content (i.e., whole food standard recovery nutrition recommendation *vs*. volume matched higher nutrient density of reconstitute recovery supplement) on gastrointestinal and immune status, the finding suggest whole food dairy milk poses a lower gastrointestinal intolerance, despite equal outcomes for other recovery optimisation markers.

### Gastrointestinal Responses and Acute Exercise Recovery Beverage

It is recommended and is a common practice for athletes to consume food/s and fluid/s in the early stages after exercise cessation (i.e., training and/or competition). The diverse availability of manufactured recovery products, especially beverages, either as whole-food (e.g., dairy milk) or supplementation (e.g., dairy based supplement powder) form, appears to promote diverse nutrient density intakes, representing an ideal to over-load of nutrient provision. Considering exercise stress compromises gastrointestinal functional activities (i.e., motility, digestion, and absorption) and prompts GIS, as a result of exercise-induced gastrointestinal syndrome (10), it has been previously unknown how the provisions of differing nutrient density recovery beverages impacts on post-exercise gastrointestinal status. Therefore, the nutritional quantity and quality provided during the exercise recovery period may affect how an athlete tolerates the recovery nutrition provided (6, 7, 14). Participants in the current study did not present lactose intolerance or dairy protein allergies, yet a clinically significant rise in breath H_2_ was observed 1.5 h after consumption of both beverages, with a significantly greater peak observed after consumption of the MBSB (Figure 2A) (43). Figure 2B highlights the large individual variation, as the participants recording the highest peak breath hydrogen concentrations (84–121 ppm) were those who experienced diarrhea on the MBSB trial, two of whom also experienced diarrhea on the CM trial. The remaining participants experienced mild or no lower-GIS on either trial, despite clinically significant malabsorption. Asymptomatic malabsorption (i.e., >10–20 ppm) has been observed amongst healthy individuals (i.e., no known gastrointestinal disease or disorder), following consumption of lactose (50 g), and sucrose (<100 g), at rest (44, 45). It is although possible that the observed carbohydrate malabsorption in the current study was exacerbated by neuroendocrine and circulatory derived exercise induced gastrointestinal syndrome, leading to oversaturation of epithelial transporters (10, 46, 47). The current study provides evidence of individual, nutritive, and exercise-induced factors may separately and interactively contribute to malabsorption of dairy based exercise recovery beverages. However, further research is required to clarify underlying mechanisms and possible strategies for remediation.

### Immune Responses and Acute Exercise Recovery Beverage

Athletes commonly cite using sports food products to prevent microorganism-borne illness and infection (48). Dairy milk beverages have been reported to prevent the exercise-induced immunodepression commonly observed after strenuous exercise, and interestingly one recent study showed an acute boost in immune functional responses (i.e., 85% increase *in-vitro* bacterially-stimulated neutrophil elastase release) in the exercise recovery period; although dairy milk beverages are not promoted as immunostimulants (6). This benefit appears to be specific to dairy beverages, as opposed to non-dairy beverages with equivalent or similar nutritional composition. This phenomenon has been attributed to the potential combination of insulinaemic and calcaemic effect of dairy milk, on priming neutrophil functional responses (e.g., suppressing desensitization, enhancing phagocytosis and elastase release efficiency) (6, 7, 49–52). The current study failed to demonstrate such an immunostimulation, or even prevent the commonly observed exercise-induced depression (19% reduction in the current study) of *E.coli* LPS challenged neutrophil elastase release with either beverage. However, it is important to highlight that the reported reduction in *in-vitro* bacterially-stimulated neutrophil elastase release did not reach significance compared with pre-exercise baseline level. This may be likely due to the 2 h HIIT exercise model providing a low exercise stress load than previous exercise models reporting ~30% reduction in neutrophil function with running at ≥70% *V*O_2max_ in temperate ambient conditions (5–7, 9). Nevertheless, the absence of a preventative effect is likely associated with delay in delivery of the beverage, as previously observed (5). More research is needed to determine if immediate provision of carbohydrate and protein > 1.2 g/kg BM and 0.4 g/kg BM, respectively, and the associated heightened insulin response, will further enhance immune functional responses, and whether this enhancement is at all beneficial (e.g., reduced microorganism-borne illness or infection risk, intestinal originated bacterial endotoxin systemic translocation clearance, and/or exercise associated tissue adaptions) from a translational perspective, considering the *in-vitro* methodologies used in the current study and the interaction of immune response components *in-vivo* (53).

It is well-established that exercise stress has the capability of inducing a systemic inflammatory response (54), which appears to be proportional to the exertional load and exacerbated by external factors (e.g., environmental conditions- heat) (22, 34). Evidence from the recent scientific literature suggests that exercise stress up to 3 h in duration, with or without additional heat stress, promotes modest increases in systemic inflammatory profile, characteristic of none to small increases in pro-inflammatory cytokine markers (i.e., TNF-α and IL-1β), none to modest increases in systemic response cytokine markers (i.e., IL-6 and IL-8), and modest to large increases in anti-inflammatory cytokine markers (i.e., IL-10 and IL-1ra) (6, 22, 26, 34, 42, 55–57). For example, 2–3 h running at ~60–70% *V*O_2max_ consistently results in <10 pg/ml pre- to peak post-exercise (0–4 h measurement period) increase in plasma pro-inflammatory and response cytokine concentrations. However, substantially greater (e.g., >20 pg/ml) systemic anti-inflammatory cytokine responses are seen, after such exercise loads, suggesting anti-inflammatory cytokine markers (e.g., IL-10 and IL-1ra) are more sensitive to exercise induced changes. Nevertheless, it is important to note that the magnitude of systemic inflammatory cytokine responses to such exercise loads are of little clinical significance (e.g., comparable to cytokine profiles associated with clinical sepsis) (58, 59), and only with an extreme exercise load (e.g., ultra-endurance exercise) does systemic inflammatory cytokine responses appear to reach clinical relevance (55, 56). In the current study, there was no substantial systemic inflammatory cytokine response, suggesting 2 h HIIT creates no to minimal consequence to systemic inflammatory status. It is reported that the translocation of bacterial endotoxins from the lumen into circulation contributed to the systemic inflammatory response peaking at the cessation of exercise (10, 54). In the current study there was no substantial evidence of intestinal epithelial injury or increases in circulating sCD14 or LBP (i.e., indirect markers for luminal translocated bacterial endotoxin). Therefore, it is not surprising that systemic inflammatory responses were minimal. It is also suggested that certain foods have the propensity to acutely alter systemic cytokine profile in the post-prandial period (60). In the current study, inflammatory cytokines were unaltered by the recovery beverage intervnetion, with the exception of IL-1β, which was significantly greater in MBSB at 4 h recovery compared to CM. It is unclear why this response was seen following consumption of the MBSB, but links to some inflammatory aspect within product development and provisions should not be overlooked. It should also be noted that the magnitude of difference in IL-1β between MBSB and CM (i.e., 0.7 pg/ml) appears to be of little clinical relevance, as indicated by the lack of trial differences in the SIR-profile.

### Muscle Glycogen Resynthesis and Acute Exercise Recovery Beverage

Replenishment of skeletal muscle glycogen stores through high carbohydrate intake is a primary goal of acute recovery nutrition, especially if consecutive bouts of strenuous exercise bouts are programmed in the short term (e.g., same or following day) (3), which is dependant of gastrointestinal patency (e.g., intake behavior linked to tolerance and GIS, ingested food-fluid motility, digestion and absorption) and potentially immune responses associated with systemic pathogenic (e.g., luminal originated bacteria and/or bacterial endotoxin) and tissue injury (e.g., cell damage debris) clearance (10, 11). In the current study, an intake of 1.2 and 2.2 g/kg BM of carbohydrate on CM and MBSB trials, respectively, failed to initiate muscle glycogen replenishment processes 90 min after consumption. Blood glucose and insulin responses on both trials indicate glucose absorption, systemic availability, and uptake by insulin-sensitive tissues (Figures 3A,B). However, phosphorylation of GSK-3β remained unchanged, suggesting a limitation of glucose uptake at the level of skeletal muscle, and not at the gastrointestinal level. Therefore, impaired glucose uptake at the sarcolemma, and disposal of glycogen within the skeletal muscle cell are likely to have limited resynthesis of skeletal muscle glycogen. Skeletal muscle-damaging eccentric exercise protocols have provided evidence of impaired rates of muscle glycogen resynthesis, associated with compromised cell structural integrity, reduced translocation of GLUT-4 to the skeletal muscle plasma membrane, and reduced insulin sensitivity (61). Given the sampling time-frame in the current study, it is unclear if greater carbohydrate intakes on the MBSB enhanced glucose disposal and glycogen synthesis beyond 2 h post-exercise. Further research is required to verify these findings and examine possible mechanisms.

### Protein Synthesis Expression to Acute Exercise Recovery Beverage

Following prolonged, strenuous exercise, provision of adequate protein is required to generate a net positive protein balance (62), which is also dependant on gastrointestinal patency and potentially immune responses. In the current study, CM 30 (6) g protein (derived from fresh cow's milk protein) and MBSB 63 (14) g protein (derived from non-fat milk solids and whey protein concentrate) equally increased phosphorylation of mTOR and Akt. Findings from the current study are supported by previous literature demonstrating maximal myofibrillar fractional synthetic rate following endurance exercise occurred with 23–30 g milk protein intake, with no further benefit at an intake >45 g (63, 64). Whey protein derived from dairy milk has been identified as a superior protein for stimulating muscle protein synthesis owing to the rapid digestibility and absorption kinetics. Digestion and absorption of protein within the whole milk matrix and with carbohydrate co-ingestion is delayed compared to isolated whey, casein or soy protein, however this does not appear to diminish rates of muscle protein synthesis provided adequate overall protein (i.e., >23 g) is consumed (65, 66). Quantification of myofibrillar and mitochondrial fractional synthetic rate, and the corresponding aminoacidaemia and leucinaemia, following consumption of dry and liquid dairy proteins is warranted to expand on these findings.

### Hydration Status to Exercise Recovery Beverage

Considering hydration status (e.g., hypohydration vs. euhydration) has been shown to impact gastrointestinal patency and immune functional responses (i.e., *E. coli* LPS challenged neutrophil elastase release) (6), it seems plausible that an enhanced rehydration beverage would also benefit gastrointestinal and immune status. Beverages with a greater nutrient density have been shown to aid fluid retention via delaying gastric emptying (i.e., avoiding excessive water dumping along the intestine), enhancing intestinal water absorption and circulatory water retention through the osmotic properties of present nutrients (67). Indeed, flavored and unflavoured dairy milk products have been shown to enhance fluid retention compared to equal intakes of carbohydrate-electrolyte beverages with a lower energy density, and equal or greater sodium concentrations (16, 18, 68, 69). For example, following mildly dehydrating exercise (i.e., <1.8% BM loss), 150% replacement of BM losses with skim milk (i.e., 5 g/100 ml of carbohydrate and 3–4 g/100 g of protein, and 17–58 mmol/L of sodium) resulted in a more positive net fluid balance, and corresponding lower urine output and greater P_Osmol_, compared to isovolumetric intake of water or carbohydrate-electrolyte beverage (i.e., 4–6 g/100 ml of carbohydrate and 21–23 mmol/L of sodium) (18, 68, 69). In the current study, greater fluid retention was observed after consumption of the reconstituted MBSB, compared to CM. Globally, these findings suggest that the nutrient density of the beverage exerts a greater osmotic effect beyond that of sodium concentration (70). However, fecal losses occurred in the CM (*n* = 2) and MBSB (*n* = 5) trials, with participants reporting diarrhea on both trials. Estimated fecal losses per individual were greater on MBSB [705 (271) g], compared to CM [486 (79)], however it was not possible to quantify fecal water losses *per se*. Therefore, it is likely that the equation described for fluid retention will produce an overestimate of the true value. Importantly, exercise-induced fluid losses were mild (i.e., <2%), and euhydration was maintained throughout the prescribed exercise protocol. These findings would suggest that when mild fluid losses occur, there is a threshold to the concentration and volume of nutritive beverages that can be consumed before detrimental outcomes and excessive fluid losses occur, even for lactose tolerant individuals.

## Conclusion

Consumption of a flavored whole food dairy milk and a flavored reconstituted dairy milk based supplement beverage both increased activation of anabolic signaling (i.e., p-mTOR and p-Akt) and restored hydration status. Neither beverage increased muscle glycogen stores, nor immune functional responses (i.e., *in-vitro* bacterially-stimulated neutrophil elastase release), likely associated with impaired skeletal muscle cell structural integrity and delayed feeding, respectively. Both beverages resulted in clinically significant malabsorption of the recovery beverage, however the degree of malabsorption was significantly greater after consumption of the MBSB, and was associated with a trend toward significantly greater gut discomfort. The observed differences in the assimilation of recovery nutrition and acute recovery outcomes did not translate to differences in performance outcomes the following day. Flavored whole food dairy milk therefore provides a cheaper and more practical alternative to specially formulated reconstituted dairy milk based supplementation, and poses a lower risk of nutrient malabsorption and associated GIS.

## Data Availability Statement

The raw data supporting the conclusions of this article will be made available by the authors, without undue reservation.

## Ethics Statement

The studies involving human participants were reviewed and approved by Monash University Human Research Ethics Committee. The patients/participants provided their written informed consent to participate in this study.

## Author Contributions

RC was the chief investigator. IR was the primary investigator of the research project and contribute toward the original research idea. RC, IR, JP, and LB contributed toward development of the experimental design. IR and RC contributed to the analysis of the raw data and prepared the original draft manuscript. PD, AG, JP, and LB contributed toward data collection, sample collection, and analysis. All authors contributed to the review, final preparation of the manuscript, read, and approved the final manuscript.

## Conflict of Interest

The current study was supported by Lion Dairy & Drink Australia Pty Ltd, as part of the Monash University Food & Dairy GRIP. The funder was not involved in the development of the experimental protocol, data collection, analysis or interpretation of results. No restrictions were placed on the reporting of findings.
